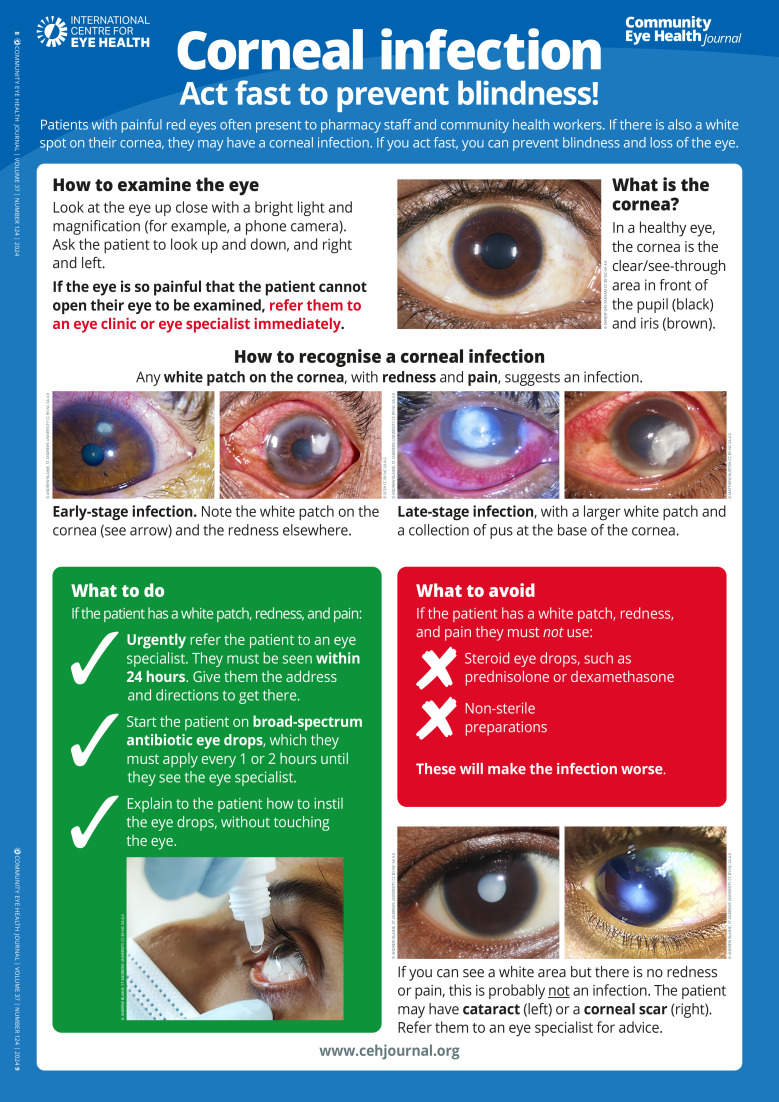# Corneal infection: Act fast to prevent blindness!

**Published:** 2025-01-31

**This article features a poster on the diagnosis and management of corneal infection.**

**Figure:**